# When Phosphatases Go Mad: The Molecular Basis for Toxicity of Yeast Ppz1

**DOI:** 10.3390/ijms23084304

**Published:** 2022-04-13

**Authors:** Antonio Casamayor, Joaquín Ariño

**Affiliations:** Institut de Biotecnologia i Biomedicina & Departament de Bioquímica i Biologia Molecular, Universitat Autònoma de Barcelona, 08193 Cerdanyola del Vallès, Spain; antonio.casamayor@uab.cat

**Keywords:** protein phosphatases, protein overexpression, transcriptomics, phosphoproteomics, intracellular signaling, pH homeostasis, *Saccharomyces cerevisiae*

## Abstract

The fact that overexpression of the yeast Ser/Thr protein phosphatase Ppz1 induces a dramatic halt in cell proliferation was known long ago, but only work in the last few years has provided insight into the molecular basis for this toxicity. Overexpression of Ppz1 causes abundant changes in gene expression and modifies the phosphorylation state of more than 150 proteins, including key signaling protein kinases such as Hog1 or Snf1. Diverse cellular processes are altered: halt in translation, failure to properly adapt to low glucose supply, acidification of the cytosol, or depletion of intracellular potassium content are a few examples. Therefore, the toxicity derived from an excess of Ppz1 appears to be multifactorial, the characteristic cell growth blockage thus arising from the combination of various altered processes. Notably, overexpression of the Ppz1 regulatory subunit Hal3 fully counteracts the toxic effects of the phosphatase, and this process involves intracellular relocation of the phosphatase to internal membranes.

## 1. Introduction

Overexpression of a given protein can cause harm to the cell for multiple reasons. For instance, intrinsically disordered regions in overexpressed proteins display a tendency to produce protein aggregation by making promiscuous molecular interactions [1]. The stoichiometric balance among members of macromolecular protein complexes can also be altered [2]. Other possible causes can be the inappropriate liquid phase separation forced by the increased protein concentration [3], the disruption of the regulation of specific pathways, or the excessive draining of the cell resources to build and/or transport proteins [4]. A considerable quantity of cellular energy should be invested to reduce the excess of proteins, which leads to a delay in the cell cycle in yeast cells [5]. The cellular resources required for protein degradation denote the importance of the proper protein dosage, which seems specific for each type of protein and might be relevant for subunits of protein complexes or proteins involved in cellular signaling [5,6]. Among the possible causes of naturally occurring protein overexpression, aneuploidy is one of the most studied [7]. In yeast, aneuploidy leads to changes in protein expression that result in slow growth and oxidative and metabolic stress [8].

The *Saccharomyces cerevisiae* Ppz1 and Ppz2 enzymes are type 1-related Ser/Thr protein phosphatases. They are composed of a C-terminal catalytic domain ~60% identical to the yeast PP1 catalytic subunit (PP1c) Glc7 and contain a long N-terminal extension of about 350 residues [9,10]. Whereas Ppz1 and Ppz2 catalytic domains are very conserved (86% identity), their N-terminal extensions are much more divergent (43% identity). Remarkably, PPZ enzymes are found only in fungi [11], and the fact that Ppz1 has been related to virulence in some human pathogenic fungi such as *Candida albicans* [12] and *Aspergillus fumigatus* [13] has raised significant attention on these enzymes in recent years.

In *S. cerevisiae*, the role of Ppz1 is more prominent than that of Ppz2. Ppz1 regulates monovalent cation homeostasis in two ways: by inhibiting K^+^ uptake in both Trk-dependent and independent manner [14,15] and by repressing the expression of the Na^+^/ K^+^-ATPase encoded by the *ENA1* gene [16,17]. A role for Ppz1 in translation fidelity has also been reported through the regulation of Tef5, a subunit of translation elongation factor EF-1A [18]. Similarly, Ppz1 appears to be involved in the dephosphorylation of ubiquitin at S57, within the context of the regulation of endocytic trafficking and ubiquitin turnover [19] and in the dephosphorylation of the arrestin Art1 in a process linked to the endocytosis of the methionine transporter Mup1 [20].

Ppz1 function is regulated in vivo by two proteins that act as inhibitory subunits, Hal3 and Vhs3. Both proteins bind to the catalytic domain of the phosphatase and inhibit its enzymatic activity [21,22,23], although the role of Hal3 appears more prominent in vivo. Hal3 and Vhs3 are moonlighting proteins; they function not only as Ppz1 inhibitors but also, together with Cab3, as components of the enzyme phosphopantothenoylcysteine decarboxylase (PPCDC), which catalyzes a key step in coenzyme A biosynthesis [24].

Early evidence suggested that Ppz1 function must be subjected to tight regulation and that excessive Ppz1 activity was detrimental to the cell. It was reported that when *PPZ1* was expressed from an episomal plasmid, from either its own native promoter or the strong inducible *GAL1-10* promoter, growth was severely or fully blocked [21,25]. Lately, the work of Makanae and coworkers [26], based on the genetic tug-of-war (gTOW) strategy, revealed that *PPZ1* is one of the genes for which the cell has the lowest tolerance limit, indicating that the cell cannot endure the accumulation of the phosphatase. Accompanying *PPZ1*, this work also uncovered genes coding for protein kinases such as *TPK1*, *TPK2*, *TPK3,* or *PRK1*, as well as for the protein phosphatase *CDC14*. These results emphasized that deregulation of the protein phosphorylation status, likely leading to alteration of diverse signaling pathways, can have dramatic effects on cell growth and survival.

In this review, we will describe how work developed in the last few years and based on diverse and complementary experimental approaches has provided a clearer picture of the specific molecular basis underlying the toxicity stemming from an excess of Ppz1 activity.

## 2. The N-Terminal Extension of Ppz1 Is an Important Factor for Toxicity

As mentioned above, the *S. cerevisiae* genome encodes two PPZ paralogs, *PPZ1* and *PPZ2*, which are very closely related in sequence in their C-terminal catalytic domain (85.9% identity, 89.9% similarity). However, their N-terminal extensions are far more divergent. Early evidence showed that strong overexpression of the C-terminal catalytic moiety of Ppz1 (348 residues) was also highly toxic [25] and, more recently, it was reported that the activity of Ppz1 was a requirement for toxicity [27]. In addition, overexpression of the Ppz1 and Ppz2 inhibitory subunit Hal3 was able to fully restore normal cell growth [21]. Therefore, it was reasonable to test if overexpression of *PPZ2* would also be detrimental to cell growth. Yet, expression of Ppz2 under the same conditions used for Ppz1, driven from a *tetO*-regulatable promoter in a multicopy plasmid, showed very little effect, if any, on cell growth [28]. In fact, this lack of toxicity was not surprising since the work of Makanae and coworkers [26] revealed that yeast cells could tolerate a higher load of plasmid bearing *PPZ2* than *PPZ1*. It must be noted, though, that the levels of Ppz2 protein in the *tetO*-based experiments were significantly lower than that of Ppz1, raising the possibility that the lack of toxicity could be due to insufficient accumulation of the phosphatase. However, a hybrid version carrying the N-terminal half of Ppz1 and the catalytic domain of Ppz2 was as toxic as Ppz1 even if it was expressed at levels similar to that of native Ppz2 [28]. This indicated that the disordered Ppz1 N-terminal region is an important determinant for toxicity and that this feature is not shared by the extension found in Ppz2. In fact, the functional relevance of the Ppz1 N-terminal extension was hinted at long ago by Clotet and coworkers [25], who reported that particular deletions in this region affected the ability of the protein to rescue specific phenotypes of a *ppz1* mutant strain. Current efforts in our laboratory aim to identify the precise structural features in the Ppz1 N-terminal region that are crucial for toxicity.

## 3. *PPZ1* Overexpression Affects Protein Synthesis

It has been recently demonstrated that the excess of Ppz1 activity impairs protein synthesis, probably at the initiation step. This might be due to a possible role of Ppz1 in regulating ribosome biogenesis and function since the endogenous (non-overexpressed) Ppz1 protein has been co-purified with ribosomal proteins and localized in a wide range of ribosomal fractions covering the 40S and 60S ribosomal subunits, the 80S ribosome, and polysomes [27]. In addition, overexpression of *PPZ1* altered the ribosomal function, causing an important reduction in the polysome content, which was normalized when the Ppz1 inhibitor Hal3 was overexpressed. We demonstrated that a relatively delayed effect of Ppz1 overexpression led to an increase in the phosphorylation of the eIF2α translation initiation factor at Ser51, likely mediated by the Gcn2 protein kinase [27]. Phosphorylation of eIF2α at its Ser51 is one of the inhibitory mechanisms that repress translation, impairing the regeneration of GTP-bound eIF2 and thus the formation of the ternary complex with GTP and the initiator Met-tRNA_I_ required for translation initiation. Another inhibitory mechanism is the sequestering of eIF4E by the eIF4E-binding proteins Eap1 and Caf20, which interferes with the formation of the closed-loop complex and represses translation. Our observation that deletion of *GCN2* improves, to some extent, the growth of cells overexpressing Ppz1, but lack of Caf20 or/and Eap1 does not, suggests that the excess of Ppz1 affects the formation of the ternary complex rather than that of the closed-loop complex. Nevertheless, in a recent phosphoproteomic study using the yeast strain ZCZ01, in which the genomic *PPZ1* copy is controlled by the strong *GAL1-10* promoter, we found Eap1 T284 dephosphorylated after 1 h of Ppz1 induction, and it remained in this state up to 4 h [29]. Although about 30 phosphorylated residues have been detected in Eap1, according to post-translational modifications in Saccharomyces Genome Database (SGD, https://www.yeastgenome.org, accessed on 27 January 2022), this is the first report describing the phosphorylation of T284 and, therefore, the consequences of this phosphorylation are unknown.

Further evidence about the effect of an excess of Ppz1 activity on protein synthesis came from the identification of changes in the phosphorylation state of diverse proteins involved in this process [29]. As shown in Table 1, 5 proteins required for translation initiation were found hyperphosphorylated, while residues in 10 proteins were significantly dephosphorylated, including those in Rps6, a conserved component of the small (40S) ribosomal subunit [29]. Regarding Rps6, overexpression of Ppz1 triggered fast dephosphorylation of S232 and S233 (decrease to 45% after 1 h), which continued to decline until very low levels after 4 h (16%) [29]. It is known that phosphorylation of S232 and S233 in response to nutrients, among other stimuli, occurs in a TORC1- and TORC2-dependent manner [30,31]. Phosphorylation of these sites is mediated by the Sch9 and Ypk3 protein kinases [30,32,33], and it has been reported that Glc7, together with its regulatory subunit Shp1 dephosphorylate S232 and S233 [30]. Moreover, expression of an unphosphorylatable S232/S233 mutant version of Rps6 impeded cell growth with an almost 30% decrease in the cells proliferation rate and, although it did not affect the polysome to 80S monosome ratio, a defect in the biogenesis of the 40S small subunit was observed [30]. Therefore, it is plausible that aberrant dephosphorylation of Rps6 could contribute to Ppz1 toxicity.

Dot6 is one of the transcriptional repressors that, when phosphorylated by the TORC1 substrate Sch9 kinase in response to nutrient limitation, releases its repressor effect on genes coding for ribosomal proteins (RP) and proteins required for ribosomal biogenesis (RiBi) [34]. Interestingly, *DOT6* came out in a screen looking for multicopy suppressors of growth arrest caused by the excess of Ppz1 [27]. Seventy-two phosphorylation sites have been described in Dot6 (SGD), and it has been shown that mutation of five serine residues in the RxxS motif to alanine strongly reduced both its phosphorylation by Sch9 and the transcription of proteins required for protein synthesis (RP and Ribi) [34]. We have identified in some experiments that the excess of Ppz1 triggers the dephosphorylation of several residues, including three of these five serines (S247, S282, and S313) [29]. We cannot exclude that when overexpressed, Ppz1 might dephosphorylate Dot6, thus promoting the down-regulation of genes coding for RP and Ribi. This hypothesis might explain why, together with *DOT6*, the above-mentioned multicopy suppressor screen yielded *NOC2,* important for ribosome biogenesis, as well as several genes coding for ribosomal proteins (*RPS6A*, *RPS15*, *RPP2*, *RPL37A,* and *RPL37B*) [27].

## 4. An Excess of Ppz1 Interferes with Normal Adaptation to Low Glucose

The effects of mild expression of Ppz1 in carbon sources other than galactose were investigated by placing the *PPZ1* ORF under the control of the doxycycline-repressed promoters *tetO_2_* and *tetO_7_*, present in strains MLM03 and MLM04 [27]. Remarkably, the deleterious effect on cell growth under these circumstances was only evident in medium containing low glucose concentrations but not in the presence of 2% glucose. The toxic effect was even more dramatic when cells were grown in other carbon sources, such as galactose, wherein growth was completely abolished even with 2% of the sugar in the medium [27]. These results suggested that the defect in cell proliferation induced by increased levels of Ppz1 was, to some extent, dependent on the availability of glucose.

**Figure 1:**
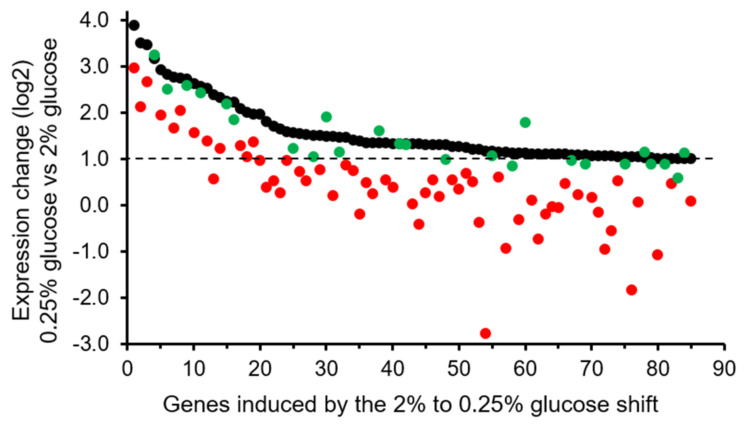
Expression changes for the set of 85 genes found induced by the shift from medium containing 2% glucose to medium containing 0.25% glucose (black circles). The expression changes for each of these genes in response to the shift when *PPZ1* was overexpressed are also plotted for genes affected (red circles) or unaffected (green circles) by the overexpression of the phosphatase (unpublished results, data can be retrieved from Gene Expression Omnibus (GEO) under accession code GSE199793). A dotted line is drawn to better distinguish upregulated genes.

To further investigate this possibility, we have used mRNA-Seq to determine the transcriptional response triggered by the shift from high to low glucose in normal cells and in cells overexpressing *PPZ1* from the *tetO_7_*-driven multicopy pCM190 plasmid. Once the expression of *PPZ1* was induced for 3.5 h, the transcriptional response after 1 h of the shift from 2% to 0.25% glucose was determined in both strains. Assuming only those genes whose mRNA increased at least two-fold in the presence of 0.25% glucose, our mRNA-Seq data revealed that 85 genes were induced by low glucose in cells containing the empty pCM190 vector (unpublished results). As expected, the most over-represented Gene Ontology (GO) category for these genes, according to the YeastMine tool [35], was carbohydrate metabolic process (GO:0005975) with 22 genes (*p*-value 7.4 × 10^-9^). Interestingly, the expression level for this set of 85 genes was clearly lower in many cases in cells overexpressing Ppz1 than in cells with the empty plasmid (average of 3.00-fold vs. 1.66-fold induction) (Figure 1). To identify the possible role of the excess of Ppz1 in the low glucose-induced transcription, we classified as Ppz1-dependent those genes whose expression levels were reduced at least 30% in cells overexpressing *PPZ1*. We found that the expression of 25 genes was not affected by high levels of Ppz1, whereas 60 genes were Ppz1-dependent (Figure 1). GO analysis of this set of 60 Ppz1-dependent genes indicated that the carbohydrate metabolic process was again the most represented (19 genes; *p*-value 3.19 × 10^-8^). No significant GO categories were found among the set of 25 genes unaffected by an excess of Ppz1. These results suggested that overexpression of Ppz1 under the tetO promoter from the pCM190 plasmid does impair the normal transcription adaptation to conditions in which glucose becomes limiting by interfering with the proper activation of a set of genes mainly involved in carbon metabolism.

To further analyze our transcriptomic data, the Yeastract+ portal [36] was used to identify the transcription factors functionally associated with the two above-mentioned gene populations and the possible differences among them. Table 2 shows that several repressors known to downregulate gene expression in the presence of glucose, such as Mig2, Mig1, Nrg1, and Nrg2, were highly enriched for genes induced in normal cells. In contrast, they were much less prominent in cells overexpressing Ppz1, indicating a weakening of the response induced by glucose scarcity. This list also highlighted the possible role of Mig1/Mig2 transcriptional repressors in this differential response.

**Figure 2:**
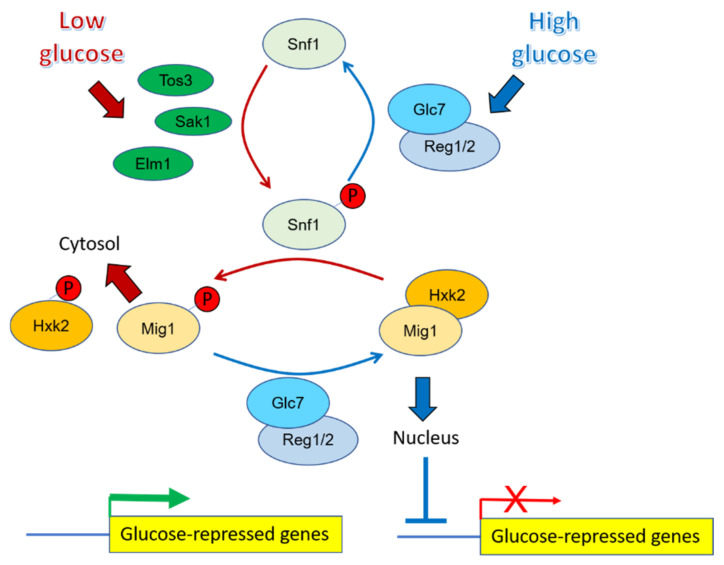
Simplified representation of known phosphorylation mechanisms regulating the transcriptional repressor Mig1 under high and low glucose conditions. The model has been drawn based on data extracted from references [37,40].

Nuclear localization of the Mig transcriptional repressors is controlled by phosphorylation and by the interaction with Hxk2. In the presence of high glucose, unphosphorylated forms of Mig1 and the major glucose kinase Hxk2 localize to the nucleus and interact with each other, allowing repression of transcription of genes required for use of other carbon sources (Figure 2). Under glucose depletion conditions, however, the Snf1 kinase is activated by phosphorylation at T210 and, in turn, phosphorylates Mig1 at S311. Under these conditions, Snf1 also phosphorylates Hxk2 at S15, thus preventing its nuclear localization and its interaction with Mig1, promoting the exit of both proteins from the nucleus and releasing their target genes from transcriptional repression [37,38,39]. Dephosphorylation of Mig1 S311 and Hxk2 S15 was attributed to the activity of the Glc7-Reg1 complex [37]. Notably, phosphoproteomic experiments demonstrated fast and sustained dephosphorylation of Mig1 at S311 and of Hxk2 at S15 when *PPZ1* expression was induced, as well as concomitant dephosphorylation of Snf1 T210. These changes were accompanied by substantial retention of Mig1 in the nucleus [29].

Snf1 is also dephosphorylated and inactivated by the Glc7-Reg1 complex, and, as a result, Snf1 can no longer phosphorylate the Mig transcriptional repressors [40,41]. About 60 phosphorylated residues have been detected in Reg1 according to the data collected by the SGD [42]. Residues in its N-terminal segment (1–400 aa) were phosphorylated under glucose-limiting conditions in an Snf1-dependent manner, thus regulating the Reg1-Bmh1/2 interaction [40,43], and this N-terminal region acts as an in vitro substrate for Ppz1 [44]. It is suggestive that our phosphoproteomic data show that Reg1 is significantly dephosphorylated (about 50%) in the N-terminal residues S346 and S349 after 1 h from the induction of the Ppz1 overexpression, remaining dephosphorylated for at least 4 h [29]. The relevance of these phosphorylation sites of the Reg1 N-terminal segment in the glucose repression process is still unknown. Taken together, these data show that the excess of Ppz1 might disrupt the proper adaptation of yeast cells to conditions of low glucose by acting on several targets of the pathway involved in the regulation of the Mig transcriptional repressors. This may proceed by affecting physiological Ppz1 targets or by interfering with natural substrates for Glc7.

Based on these results, it could be argued that disruption of the normal mechanism for adaptation to glucose scarcity could be a main reason for the growth defect caused by higher-than-normal Ppz1 levels. However, while this could be a limiting factor for mild overexpression of the phosphatase, we have observed, using a ZCZ01 derivative (strain MAC01), in which expression from the *GAL* promoter is triggered by β-estradiol, that cell growth is completely blocked even in the presence of 2% glucose [45]. Since strain MAC01 expresses Ppz1 at levels equivalent to that of strain ZCZ01, it can be postulated that the surplus of Ppz1 activity affects additional signaling pathways required for one or more essential cellular functions.

## 5. Ppz1 Overexpression Affects Diverse Signaling Pathways

Genome-wide transcriptomic and phosphoproteomic analyses of the short-term response to Ppz1 overexpression using the ZCZ01 strain [29] revealed a widespread impact on the yeast cell. Nearly 1300 genes (about 20% of the genome) suffered a significant modification in their expression. These included cyclins *CLN1*, *CLN2*, *CLB1*, and *CLB5*, which are repressed, consistently with the previously reported halt in the G1 phase caused by excess of Ppz1 [27,46]. Similarly, the mRNA levels of many genes involved in ribosome biogenesis showed a very early decline (30 min after Ppz1 induction). Strikingly, the entire Bas1-Bas2-dependent ADE pathway, required for de novo purine biosynthesis, was also repressed. Metabolomic analyses revealed a marked increase in ATP and GTP levels, as well as in the adenylate pools and in the adenylic energy charge, thus suggesting that the transcriptomic effect could be due to a negative feedback loop caused by the accumulation of its final products. Likewise, the levels of dNTPs were also augmented. These results indicated that the halt in growth caused by high levels of Ppz1 is not due to the scarcity of building blocks for DNA synthesis or a lack of energy.

Cells overexpressing Ppz1 suffer oxidative stress. This conclusion was deduced from the transcriptomic data (strong induction of known oxidative stress-responsive genes) and further confirmed by the determination of reactive oxygen species (ROS). Because it is well known that oxidative stress is responsible for DNA damage [47], this could explain the formation of Rad52 foci, a common response to DNA damage, observed in Ppz1-overexpressing cells [29]. In fact, it has been reported that respiration is activated in response to DNA damage, leading to increased ATP production and to elevated dNTP levels [48], which are required for efficient DNA repair and cell survival. This link may explain the effects on nucleotide metabolism described above. In addition, oxidative stress constitutes one of the cues that activate the Gcn2 kinase, leading to the phosphorylation of eIF2α and to the reduction in protein synthesis [49]. Thus, it is conceivable that oxidative stress may contribute to the above-mentioned alteration in translation found in Ppz1-overexpressing cells.

The proteomic study of changes induced by Ppz1 overexpression revealed that very few proteins became more abundant, in agreement with the reported negative effect on protein synthesis [27]. In contrast, a significant impact on the phosphoproteome was observed, with 304 unique sites showing at least a two-fold decrease in phosphorylation found in 134 different proteins. Eighty phosphosites, found in 36 different proteins, displayed at least a two-fold increase in phosphorylation, presumably as secondary effects of Ppz1 overexpression [29]. Among these proteins, a relevant number of protein kinases or phosphatases could be identified that could amplify the effects of Ppz1 overexpression on the phosphoproteome (Table 3). Interestingly, among the set of 134 proteins found dephosphorylated, a significant number (44 proteins, *p*-value: 1.23 × 10^-9^) are contained within the cell cycle process category (GO:0022402). These include three protein kinases (Kcc4, Gin4, Hsl1) that are involved in septin organization and bud emergence, a process that is blocked in cells overexpressing Ppz1 [4,5]. Several protein phosphatases (mainly their regulatory subunits) were affected in their phosphorylation state. Some of them, such as Gip2 (regulator of Glc7) and Sap155 (regulator of Sit4), were phosphorylated, while Reg1, Bni4, and Gac1 (all three Glc7 subunits) and Rts1 (subunit of Pph21/22) were dephosphorylated.

Two other protein kinases were investigated further. The first one was Snf1, whose role in response to carbon source limitation is described in the previous section. However, Snf1 has also been related to the regulation of the cell cycle. Inhibition of the Snf1 activity leads to a decrease in the expression of G1-specific genes and the accumulation of cells in the G1 phase [50,51]. A similar phenotype is obtained by expressing the Snf1-T210A non-phosphorylatable mutant, whereas slow growth and delayed G1/S transition could be normalized by the expression of the phosphomimetic Snf1 (T210E) variant. In addition, cell cycle-dependent changes in the phosphorylation state of Snf1 have been reported. The kinase dephosphorylated at T210 during the transition from G1 to S phase and phosphorylated again at the onset of DNA synthesis [52,53]. All this evidence suggests that the abnormal dephosphorylation of Snf1 T210 observed in cells overexpressing Ppz1 may contribute to its characteristic growth defect.

A second kinase whose phosphorylation state changed upon overexpression of Ppz1, in this case by increasing phosphorylation at its key T174 and Y176 residues, was the Hog1 kinase. Phosphorylation at these residues is known to activate Hog1, and such activation induces a delay in the G1-S transition, which occurs by two mechanisms: i) phosphorylation of Sic1, the cyclin-dependent kinase (CDK) inhibitor, at T173, and ii) inhibition of transcription of G1 cyclins by targeting Whi5, which acts as a repressor of G1-specific transcription, and the Msa1/2 transcription factors [54,55]. In agreement with a hypothetical role of Hog1 activation in the cell cycle blockage induced by Ppz1 overexpression, deletion of Hog1 improves the growth of cells overexpressing the phosphatase [29].

## 6. The Alteration of Monovalent Cation and pH Homeostasis Contributes to Ppz1 Toxicity

The relationship between Ppz1 and monovalent cation homeostasis was explored long ago, prompted by the finding that the *ppz1* and *ppz1 ppz2* deletion strains were highly tolerant to sodium and lithium cations [16]. This effect was initially attributed to a depression of the *ENA1* Na^+^,K^+^-ATPase gene, involved in response to salt stress [16,17], but shortly afterward, the contribution of negative regulation of potassium influx through the Trk1/Trk2 high-affinity potassium transport system was proposed [14]. Because a strain lacking the Trk1 and Trk2 transporters cannot grow in standard media unless supplemented with potassium [56], it was reasonable to assume that the growth blockage caused by an excess of Ppz1 might derive from a strong inhibition of Trk-mediated potassium influx.

However, we recently showed [57] that cells overexpressing Ppz1 do not grow even in the presence of potassium amounts able to sustain the growth of a *trk1 trk2* strain. This result clearly argued against Trk inhibition being the basis of Ppz1 toxicity. Interestingly, deletion of the gene encoding the Na^+^,K^+^/H^+^ plasma membrane antiporter Nha1 resulted in improvement of growth in Ppz1-overexpressing cells, placing again the focus on monovalent cation homeostasis. Nha1 is a housekeeping protein that exports Na^+^ and K^+^ in exchange for protons. In conjunction with the Ena1 ATPase, it enables cell growth in the presence of high concentrations of toxic monovalent cations [58,59]. This antiporter also plays a key role in the regulation of internal pH and membrane potential [58,60] and is involved in the very early response to osmotic stress [61]. Indeed, overexpression of Ppz1 promoted intracellular acidification and depletion of intracellular potassium content, and these effects were partially counteracted in cells lacking Nha1 [57]. In addition, the beneficial effect of the *NHA1* deletion vanishes when cells are grown at pH near neutrality. All this evidence suggests that high levels of Ppz1 lead to the hyperactivation of Nha1 and a subsequent exacerbated influx of H^+^ in exchange for K^+^ ions. Several pieces of evidence support this scenario: (i) Nha1-mediated potassium efflux activity in cells overexpressing Ppz1 is far higher than in control cells, and (ii) as expected, expression of native Nha1 eliminates the growth improvement derived from the *NHA1* deletion, whereas expression of mutated versions of Nha1 (such as the D77N variant), unable to mediate H^+^/cation exchange, did not.

It has been reported that Nha1 S481 is responsible when phosphorylated by an unknown kinase for binding to yeast 14-3-3 proteins and that this decreases Nha1 activity [62,63]. This is suggestive because it is known that the mutation S481A, which prevents phosphorylation of this Ser residue, significantly increases Nha1-mediated cation efflux [62]. In our recent work [29], we identified Nha1 S481 as one of the early targets for dephosphorylation in Ppz1 overexpressing cells, raising the possibility that such dephosphorylation may activate Nha1 and contribute to the growth arrest phenotype. In concordance with this hypothesis, the expression of the S481A variant in cells lacking Nha1 and overexpressing Ppz1 not only did not improve growth but actually worsened it [57].

The fact that deletion of *NHA1* did not fully normalize growth rate and K^+^ and H^+^ intracellular contents in cells overexpressing Ppz1 indicates that additional targets affected by Ppz1 overexpression and relevant for monovalent cation homeostasis must exist. Although their nature is still obscure, several pieces of evidence suggest that the essential H^+^-ATPase Pma1 could be one of them. We observed that cells overexpressing Ppz1 failed to acidify the medium properly even in cells where *NHA1* was deleted and that this effect was not due to lower-than-normal Pma1 levels [57]. This suggests that Pma1 could be inhibited in Ppz1-overexpressing cells. Such inhibition could contribute to the above-mentioned cytosolic acidification. Pma1 can be phosphorylated in vivo at multiple sites, and it has been demonstrated that this modification controls Pma1 function. Thus, phosphorylation of S911 and T912 and, to a lesser extent, of S899 has been deemed important for the activation of Pma1 [64,65]. Remarkably, we reported that all three residues were rapidly dephosphorylated upon overexpression of Ppz1 [29]. On the other hand, the ATPase activity of Pma1 is the major consumer of cellular ATP. There is plenty of evidence that a decrease in Pma1 activity either by means of pharmacological inhibition [66,67,68] or due to point mutations in the protein leading to partial defects in proton pumping [69] results in an increase in ATP levels. Such increase was also detected in parallel with Pma1 dephosphorylation in cells overexpressing Ppz1 [29], and this finding perfectly fits with the notion that Pma1 is inhibited when Ppz1 levels are high.

**Figure 3:**
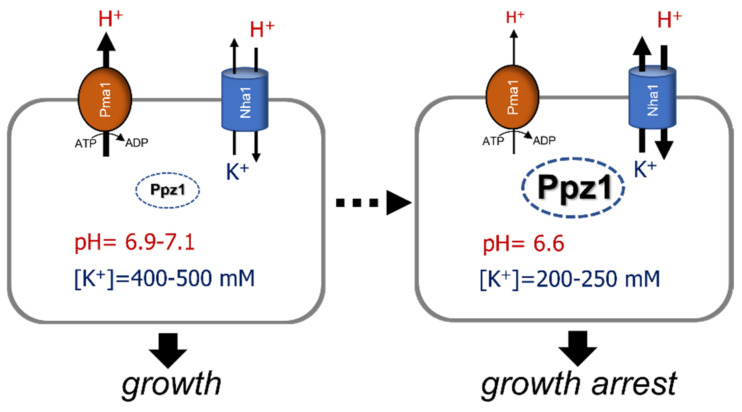
Proposed mechanism for altered K^+^ and pH homeostasis derived from Ppz1 overexpression. High levels of the phosphatase would hyperactivate the Nha1 antiporter, leading to the exacerbated entry of protons and efflux of potassium. Intracellular acidification would increase further due to inhibition of the Pma1 H^+^-ATPase. Data for cytosolic pH and cellular K^+^ content are taken from reference [57].

In conclusion, as exemplified in Figure 3, overexpression of Ppz1 alters intracellular pH and potassium levels, and these changes contribute to the growth arrest observed in these cells. The shortage of potassium would explain the striking overlap in the transcriptomic profile between Ppz1-overexpressing cells [29] and cells exposed to potassium-depleted medium [70]. Such overlapping includes genes required for ribosome biogenesis or key components in cell cycle progression, such as diverse cyclins.

## 7. Hal3: Just an Inhibitor of Ppz1 Activity?

As mentioned above, the role of Hal3 in salt tolerance and cell cycle regulation [71,72] was explained by its identification as a protein able to bind to Ppz1 at its C-terminal catalytic domain and inhibit its phosphatase activity [21,46]. However, in spite of considerable effort, the molecular basis for this inhibitory mechanism is not fully understood [73,74,75,76,77,78].

In agreement with the inhibitory role of Hal3 on Ppz1 function, expression of Hal3 from a multicopy plasmid fully restored normal growth in cells overexpressing Ppz1 either from its own promoter or from the strong *GAL* promoter [21,27]. Ppz1 is mainly located at the cell periphery, likely interacting with the plasma membrane by means of its N-myristoylated Gly2 [20,25,28,79,80]. Consequently, a G2A Ppz1 variant is fully cytosolic. Thus, the simplest scenario was that Hal3 interacts with the excess of Ppz1 and inhibits its phosphatase activity, thus avoiding harmful dephosphorylation of key cellular targets. However, we have found very recently [81] that episomal expression of Hal3 in cells overexpressing Ppz1 triggers the translocation of the phosphatase from the plasma membrane to intracellular structures that could be identified as the vacuolar membrane and, less often, the endoplasmic reticulum. Colocalization experiments showed that Hal3 accompanies Ppz1 in these intracellular locations.

Translocation of Ppz1 to internal membranes is a crucial event for the ability of Hal3 to counteract Ppz1 toxicity. Indeed, mutation of the *VPS27* gene aggravated the cell growth defect even when Ppz1 was overexpressed at moderate levels and greatly impaired the ability of Hal3 to normalize the growth of these cells [81]. *VPS27* encodes a component of the ESCRT-0 complex required for various cellular functions, including the trafficking of membrane proteins to the vacuole for degradation [82]. Interestingly, overexpression of *SPO20*, coding for a meiosis-specific subunit of the t-SNARE complex that mediates vesicle fusion required for the prospore membrane formation [83], partially ameliorated growth defects caused by an excess of *PPZ1* [27].

Whereas the mechanism underlying Ppz1 relocalization is still obscure, current evidence suggests that this is more an exceptional instrument to avoid Ppz1 toxicity, linked to excessive phosphatase activity, than a normal regulatory process. Relocalization of the phosphatase does not occur with a non-catalytic version of Ppz1 bearing the R451L mutation, which normally binds to Hal3, suggesting that critical toxic events must occur. The nature of these events is not known. However, as described above, overexpression of Ppz1 results in a fast and strong drop in intracellular pH [57], and this may, in turn, lead to increased Ppz1-Hal3 interaction [79]. Therefore, a reasonable hypothesis would be that the internal acidification caused by deregulation of Nha1, and perhaps Pma1, could trigger this phenomenon. While Ppz1 is directed in most cases to the vacuole, it is rarely detected in the vacuolar lumen, and the levels of the phosphatase are very high even many hours after induction [81]. Therefore, the translocation event is a safeguard mechanism that is not based on the degradation of the toxic protein. It is likely that localization at vacuolar and ER membranes occurs by means of the N-terminal myristoyl moiety since the G2A variant is not recruited to internal membranes [81] while exhibiting notable toxicity [28].

## 8. Conclusions

To sum up, higher-than-normal levels of Ppz1 cause a profound alteration in many cellular mechanisms. These include potassium and intracellular pH homeostasis, redox homeostasis and oxidative stress, halt in protein synthesis, or adaptation to carbon source scarcity. Such alterations derived from or are reflected in widespread changes in the phosphoproteome that affect key components of intracellular signaling such as the Hog1 or Snf1 kinases and likely result in the amplification of the effects. Therefore, the Ppz1 phosphatase is an excellent example of how overexpression of a given protein can drastically affect cellular homeostasis. We also described here a very specific contribution of the non-catalytic domain of Ppz1 in cellular toxicity and an unpredicted mechanism by which the Ppz1 regulatory subunit Hal3 contributes to alleviating the deleterious effects of the excess of the phosphatase.

## Figures and Tables

**Table 1:** Proteins involved in protein translation dephosphorylated and phosphorylated upon *PPZ1* overexpression [29]. P, phosphorylated; D, dephosphorylated.

Protein	Residues	Change	Protein Description
Cic1	S7, T11, S14, T15	D	Non-ribosomal protein associated with the nucleolar protein Nop7
Ded1	S535, S539, S572, S576	P	DEAD-box RNA helicase that associates with the eIF4F subunits
Eap1	T284	D	eIF4E-associated protein that competes with eIF4G for binding to eIF4E
Gcd6	S478, S481	D	Catalytic Ɛ subunit of the translation initiation factor eIF2B
Gcd6	T531, S538	P	Catalytic Ɛ subunit of the translation initiation factor eIF2B
Nop13	S101, T105	P	Nucleolar protein found in pre-ribosomal complexes
Sui3	S112, T116, S121, S122	P	β subunit of the translation initiation factor eIF2
Rpp1A	S96	D	Ribosomal stalk protein P1α
Rps6	S232, S233	D	Conserved component of the small (40S) ribosomal subunit
Rqc1	T158, S160, S166	D	Component of the ribosome quality control complex
Rrp1	S263, S267	D	Involved in the processing of pre-rRNA and tRNA precursors
Ssd1	S152, S154, T482	D	Controls posttranscriptional gene expression through direct binding to specific sites in mRNA
Tif4631	S505, S916, S90	P	Scaffold translation initiation factor eIF4G
Ydr239c	S257, S260, Y261	D	Protein of unknown function that might bind to ribosomes
Ymr295c	S11, T13, S14	D	Protein of unknown function that might bind to ribosomes

**Table 2:** Transcription factors (TF) controlling the set of genes upregulated by low glucose according to their dependence on Ppz1 overexpression, expressed as a *p*-value ratio. Only *p*-value ratios <1.00 × 10^−3^ are shown. The analysis was performed on data deposited at GEO (acc. # GSE199793).

TF	Targeted Genes Found (Out of 60)	*p*-Value (60 Genes)	Targeted Genes Found (Out of 25)	*p*-Value (25 Genes)	*p*-Values Ratio
Mig2	30	0	13	5.90 × 10^−^^14^	0
Mig1	26	5.00 × 10^−^^15^	14	2.59 × 10^−^^11^	1.93 × 10^−^^4^
Arr1	28	3.96 × 10^−10^	13	1.32 × 10^−^^6^	3.01 × 10^−^^4^
Nrg2	10	1.42 × 10^−^^9^	4	3.32 × 10^−^^5^	4.27 × 10^−^^5^
Sok2	39	8.45 × 10^−^^9^	16	1.12 × 10^−^^4^	7.57 × 10^−^^5^
Aft1	30	1.87 × 10^−^^7^	11	2.50 × 10^−^^3^	7.49 × 10^−^^5^
Nrg1	16	2.28 × 10^−^^7^	5	5.11 × 10^−^^3^	4.46 × 10^−^^5^
Msn2	43	2.58 × 10^−^^6^	15	3.12 × 10^−^^2^	8.29 × 10^−^^5^
Cup2	18	3.26 × 10^−^^6^	5	3.29 × 10^−^^2^	9.90 × 10^−^^5^
Crz1	20	7.53 × 10^−^^6^	4	2.04 × 10^−^^1^	3.70 × 10^−^^5^
Rox1	19	1.64 × 10^−^^5^	2	6.07 × 10^−^^1^	2.71 × 10^−^^5^

**Table 3:** Residues found phosphorylated or dephosphorylated in kinases and phosphatases involved in cell signaling. Asterisks denote proteins that were not identified as significantly modified in the phosphoproteomic experiments but whose changes were confirmed by alternative experimental methods [29]. P, phosphorylated; D, dephosphorylated.

	Protein	Residue	Change	Protein Description
***Lipid kinases***				
	Pik1	S396	P	Phosphatidylinositol 4-kinase
***Protein kinases***				
	Hsl1	S1284, S1287	D	Nim1-related protein kinase; septin-binding kinase that localizes to the bud neck
	Kcc4	S892, S894	D	Protein kinase of the bud neck involved in the septin checkpoint
	Gin4	S384, S385, S483, S486	D	Protein kinase involved in bud growth and assembly of the septin ring
	Hog1 *	T174, Y176	P	Mitogen-activated protein kinase involved in osmoregulation; controls global reallocation of RNAPII during osmotic shock
	Snf1 *	T210	D	AMP-activated S/T protein kinase; complexes with Snf4p and a Sip1p/Sip2p/Gal83p family member; required for glucose-repressed gene transcription
	Alk1	S354	D	Atypical protein kinase required for proper spindle positioning, nuclear segregation, organization of formins and polarisome components in mitosis
	Ste20	S226, S228, S289	D	Cdc42-activated signal-transducing kinase; involved in pheromone response and pseudohyphal/invasive growth
	Rck2	S46	D	Protein kinase involved in response to oxidative and osmotic stress
	Tda1	S380, S383, S523, S524	D	Protein kinase of unknown cellular role
	Npr1	S353, S256, S357	D	Protein kinase; stabilizes several plasma membrane amino acid transporters by antagonizing their ubiquitin-mediated degradation
	Ipl1	S36, S38	D	Aurora kinase of chromosomal passenger complex
	Ksp1	S814	P	Serine/threonine protein kinase; associates with TORC1 and likely involved in TOR signaling cascades
***Protein phosphatases (PP)***				
	Ppz1	T171	D	Serine/threonine protein phosphatase Z
	Ppz1	S265	P	Serine/threonine protein phosphatase Z
	Ppz2	S40	P	Serine/threonine protein phosphatase Z, isoform of Ppz1
***PP Regulatory subunits***				
	Bni4	S46, S49, S500, S503	D	Targeting subunit for Glc7 protein phosphatase; localized to the bud neck, required for localization of chitin synthase III to the bud neck
	Rts1	S263, S264	D	B-type regulatory subunit of protein phosphatase 2A (PP2A)
	Gac1	T65	D	Regulatory subunit for Glc7 type 1 protein phosphatase (PP1)
	Reg1	S346, S349	D	Regulatory subunit of type 1 protein phosphatase Glc7; involved in negative regulation of glucose-repressible genes
	Gip2	T197, T198	P	Putative regulatory subunit of protein phosphatase Glc7; involved in glycogen metabolism
	Sap155	S55, S58, Y63	P	Protein required for function of the Sit4p protein phosphatase

## Data Availability

Not applicable.
